# A Short-Term Risk Prediction Method Based on In-Vehicle Perception Data

**DOI:** 10.3390/s25103213

**Published:** 2025-05-20

**Authors:** Xinpeng Yao, Nengchao Lyu, Mengfei Liu

**Affiliations:** 1 Shandong Hi-Speed Group Innovation Research Institute, Jinan 250014, China; yaoxinpeng2005@163.com (X.Y.); liumengfei_321@163.com (M.L.); 2 Intelligent Transportation Systems Research Center, Wuhan University of Technology, Wuhan 430063, China; 3 Engineering Research Center of Transportation Information and Safety, Ministry of Education, Wuhan 430063, China

**Keywords:** traffic engineering, driving risks, short-term prediction, vehicle perception, LGBM

## Abstract

Advanced driving assistance systems (ADASs) provide rich data on vehicles and their surroundings, enabling early detection and warning of driving risks. This study proposes a short-term risk prediction method based on in-vehicle perception data, aiming to support real-time risk identification in ADAS environments. A variable sliding window approach is employed to determine the optimal prediction window lead length and duration. The method incorporates Monte Carlo simulation for threshold calibration, Boruta-based feature selection, and multiple machine learning models, including the light gradient-boosting machine (LGBM), with performance interpretation via SHAP analysis. Validation is conducted using data from 90 real-world driving sessions. Results show that the optimal prediction lead time and window length are 1.6 s and 1.2 s, respectively, with LGBM achieving the best predictive performance. Risk prediction effectiveness is enhanced when integrating information across the human–vehicle–road environment system. Key features influencing prediction include vehicle speed, accelerator operation, braking deceleration, and the reciprocal of time to collision (TTCi). The proposed approach provides an effective solution for short-term risk prediction and offers algorithmic support for future ADAS applications.

## 1. Introduction

An ADAS serves to provide drivers with warning information and assist in vehicle control when encountering driving risks. By enhancing drivers’ risk perception, it effectively reduces the likelihood and severity of collisions. This system operates by utilizing on-board sensors to collect driving-related data and predict whether selected risk indicators have surpassed a predefined threshold. However, by the time the warning system detects a risk, the vehicle has already been exposed to potential danger. Hence, the ability to accurately predict risks shortly before they manifest would allow for sufficient response time for the driver or safety systems. This aspect holds substantial importance for ensuring driving safety [1].

The first step in predicting risks is to identify them. Driving risk encompasses both the probability and severity of a vehicle collision, which is evident through actual collisions or near-collisions. By utilizing real-time traffic data and analyzing and mining data prior to risk occurrences, risk identification can effectively prevent traffic accidents [2]. Different data sources necessitate distinct risk identification methods. Currently, driving risks are primarily identified and predicted using two types of data sources: simulated driving data and natural driving data. Simulators enable the collection of driver behavior data in highly controlled environments, often utilized for researching specific scenarios and dangerous driving behaviors. Risk events can be artificially introduced in simulators [3], although differences between the simulator and real-world scenarios can impact risk model parameters and thresholds. On the other hand, natural driving data overcome the limitations associated with data collection mentioned earlier. The data are collected through various sensors installed in the vehicle, providing a realistic representation of driving data disturbances and parameter variations during risk occurrences [4]. However, natural driving data sets lack actual collision events, and obtaining a substantial number of real collision events is costly. Therefore, near-collision events are commonly employed to characterize risks, with many researchers employing surrogate safety measures (SSMs) to extract risk events.

SSMs play a crucial role in characterizing driving risk and enhancing the reliability and efficiency of warnings without relying on accident data. In 2008, the ANB20 (Safety, Data, and Analysis Committee) established the Alternative Safety Indicator Committee to facilitate and guide the development, application, validation, and implementation of alternative safety indicators.

In collision risk studies, researchers have adopted diverse methods to define risk, mainly focusing on acceleration, TTC, and its derivative indicators. Weng et al. [5] chose the deceleration rate to avoid crash (DRAC) as a risk measure. Wang et al. [6] regarded a situation as risky when the absolute value of longitudinal acceleration surpassed 1.5 or that of lateral acceleration exceeded 1.0. Naji et al. [7] considered a scenario dangerous if the braking deceleration was less than −0.4 or the braking pressure went above 10.0 Mpa. Xue et al. [8] used time headway (THW), time to collision (TTC), and margin to collision (MTC) for risk evaluation. There is also significant uncertainty regarding risk thresholds. For TTC, Ji et al. [9] determined a 4.471-s threshold for severe conflicts between freight vehicles on mountainous two-lane roads. Minderhoud et al. [10] suggested a typical TTC risk threshold range from 1.5 to 4.0 s. In Mobileye’s forward warning system, a warning is triggered when TTC falls below 2.7 s. To achieve a more comprehensive assessment, some scholars prefer to use a combination of indicators.

While risk identification contributes to improving driving safety to some extent, it can only identify risks when an event occurs, allowing for limited reaction time for the driver or safety assistance systems. Hence, it is essential to utilize short-term prediction methods to detect anomalies in on-board perception data during the period preceding a risk occurrence and provide early warnings in advance.

The short-term prediction of driving risk relies on timely and accurate identification of abnormal segments in on-board data prior to the occurrence of risks using predictive models. Currently, risk prediction models can be broadly categorized into statistical regression models and machine learning models.

Statistical regression models are typically based on historical statistical data and enable analysis and prediction. These models offer strong interpretability, providing valuable insights to traffic managers and drivers through model analysis. However, the predictive performance of these models is often limited. For example, Guo et al. [11] used a negative binomial regression model to predict high-risk drivers and identify significant risk factors, highlighting the impact of age, personality, and serious accident rates on collisions and near-collisions. Yang et al. [12] developed a Bayesian dynamic logistic regression model, where model parameters can be dynamically updated in real time based on collected traffic flow parameters and prior knowledge.

For instance, Chen et al. [13] constructed a random forest model using a random oversampling strategy to predict rear-end accidents in tunnel entrance sections, achieving an AUC of 0.807. Li et al. [14] developed a long short-term memory convolutional neural network (LSTM-CNN) model, combining CNN for extracting time-dependent features and LSTM for capturing temporal information changes, resulting in the highest AUC of 0.93 and the lowest false alarm rate of 12%. Shuangguan et al. [15] utilized a multi-layer perceptron model to predict the accuracy of high-risk states occurring in the next 0.7 s during following conditions, achieving an accuracy rate of over 85%. The construction of a prediction model involves key technologies such as determining the prediction window length, addressing sample imbalance, and conducting feature selection. These factors are crucial to ensuring that the model achieves optimal performance.

On the other hand, machine learning algorithms have drawn much attention in driving risk prediction for their strong learning capabilities. Zhang et al. [16] analyzed speed-related data of 56 ride-hailing drivers from Didi Chuxing. Their model with a modified loss function had 66.67% accuracy when using 10% of target data. Azadani et al. [17] collected data from 10 subjects and compared multiple risk-recognition models. Temporal convolution performed best under various conditions. Hu et al. [18] collected 9-D CAN bus signals from 20 subjects. Their integrated deep-learning model, combining improved networks and four data-enhancement methods, achieved 95.27% average accuracy. Using all enhancement methods, 3% of data could boost accuracy by 22.86%.

The length of the risk prediction window will significantly affect the prediction accuracy of the model. Weng et al. [5] used floating car data to match collision data, extracted aggregated data from 5–10 and 10–15 min before the collision, and analyzed its impact on the collision; Kim et al. [19] used DTG data to match collision events, extracting data within 1 min before the collision as dangerous traffic flow, and the five time periods 10–15 min before the collision were normal traffic flow; Lyu et al. [20] extracted conflict events from vehicle trajectory data, taking the 30–60 s before the conflict as a positive sample, and the other 30 s as negative samples; Chen et al. [21] conducted unsupervised learning through a three-layer L1/L2 NCAE (non-negativity-constrained deep autoencoder) network, and automatically updated the window size during the process of minimizing the objective function. The results showed that when the window size was 15 s, the original data information could be better preserved.

In conducting short-term risk prediction, a large number of positive and negative samples are needed for labeling and training. However, positive sample data labeled as risk are sparse. Therefore, risk prediction needs to undergo sample imbalance processing. Dealing with sample imbalance can be achieved at both the data and algorithm levels. At the data level, the most commonly used methods include undersampling, oversampling, and mixed sampling. Undersampling is to reduce the number of samples in most classes. The main methods include random downsampling and matched case controlled downsampling [22], and ENN (Edited Nearest Neighbours). Oversampling refers to increasing the number of minority samples. The main methods include random oversampling, SMOTE (synthetic minor oversampling technique), and ADAYN (adaptive synthetic sampling), SVM-SMOTE, K-means SMOTE, etc., which are improved based on SMOTE. At the algorithmic level, it is possible to adjust the learning weight of samples, increase the punishment after classifying a few categories incorrectly, or select models that are not sensitive to sample imbalances. In the process of dealing with sample imbalances, using only one method to improve the performance of the model is very limited. Appropriate methods should be selected from different perspectives based on the characteristics of data and algorithms to combine. Risk prediction also requires selecting appropriate characteristic variables and eliminating irrelevant and redundant variables can help improve model efficiency and generalization ability. In summary, each key technology of risk prediction needs to be optimally adjusted based on specific data and needs, but currently, driving risk prediction algorithms often ignore or simplify some key points.

This study aims to enhance driving safety by utilizing vehicle sensor data for early and accurate prediction of driving risks. To achieve this, a natural driving dataset is constructed through on-road experiments and data processing techniques. The study proposes a comprehensive system for short-term prediction of driving risks, which includes methods such as threshold selection for driving risk indicators, extraction of driving risk events, optimization of prediction windows, feature selection, short-term model prediction, and analysis. The novelty of this study lies in several aspects. Firstly, Monte Carlo simulation methods are employed to precisely calibrate the threshold values of risk indicators, ensuring accurate assessment of driving risks. Additionally, variable sliding windows and machine learning methods are utilized to explore the patterns of risk evolution. By incorporating feature selection techniques, the prediction performance of the model is both simplified and enhanced. Moreover, the study examines the impact of different data sources on prediction accuracy. The structure of the paper is organized as follows: Section 2 provides an introduction to the data source and the pre-processing methods employed in this study. Section 3 describes the research methods used, emphasizing their alignment with the data features. Section 4 presents the obtained results and provides an analysis of these findings. Finally, Section 5 summarizes the key insights drawn from this study.

## 2. Data Source and Preprocessing

### 2.1. Data Source

The data utilized in this study was obtained from real vehicle tests conducted in Wuhan. The experiment established a real-time data acquisition platform capable of collecting driving operation data, vehicle running characteristics, and surrounding vehicle driving status data. This platform effectively captures comprehensive information related to the driving process, including human-vehicle-road environment information. To acquire driver operation and partial vehicle motion posture information, we utilized the On-Board Diagnostic (OBD) system. Additionally, the OTXS device, equipped with inertial and GPS equipment, provided vehicle motion posture and location information. For gathering data on the forward vehicle target and the relative position of the vehicle and the lane markings, we employed the Mobileye device. Furthermore, the IBEO four-line laser radar, discreetly positioned at the front of the car, served as a redundant sensor to complement and validate the information collected by Mobileye. All data were accessed via the CAN bus and synchronized over time, resulting in an output frequency of 10 Hz. To validate subsequent risk events, two driving recorders were also installed. By employing this comprehensive data collection setup, we ensured the reliability and accuracy of the driving data used in our study.

**Figure 1:**
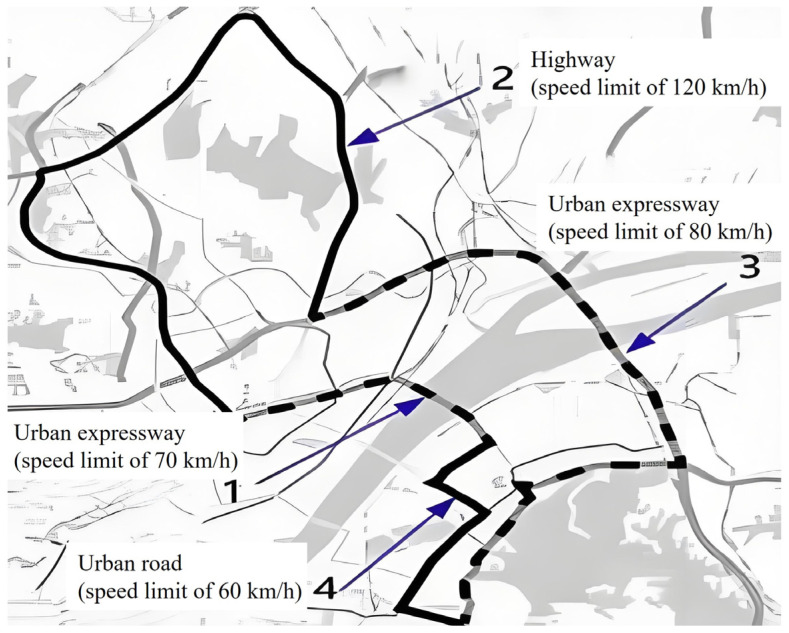
Experimental route.

The experimental route depicted in Figure 1 encompasses a 105 km stretch comprising urban roads, expressways, and highways. Segment 1 corresponds to an urban expressway characterized by a speed limit of 70 km/h. Segment 2 represents a highway with a speed limit ranging from 100 to 120 km/h. Moving on to Segment 3, it denotes an urban expressway where vehicles are subject to a speed limit of 80 km/h. Lastly, Segment 4 signifies an urban road with a speed limit set at 60 km/h.

### 2.2. Participants

The experiment recruited a total of 46 participants (25 males and 19 females) with an age range of 22–55 years (mean = 32.8, standard deviation = 8.2) and driving experience between 2–18 years (mean = 6.9). During the experiment, each driver completed two drives along the designated route, resulting in a total of 90 driving sessions. The accumulated driving data amounted to over 10,000 km, excluding two sessions that contained invalid data.

### 2.3. Data Preprocessing and Feature Extraction

The inertial navigation and OBD collected data from the real vehicle driving test are consistently stable and reliable. However, the forward target data detected by the laser radar exhibit occasional jumps and noise, although their ranging performance is excellent. On the other hand, the forward target data detected by Mobileye remain stable, but suffer from poor ranging performance and lane-offset data quality. To address the issue of device instability, a thorough evaluation of data quality for each variable is conducted, followed by average interpolation processing to address missing frame variables. Data repair is performed based on the characteristics of the sensor associated with each variable, and unrecoverable data are discarded. To overcome redundancy in the forward targets detected by both laser radar and Mobileye, a matching process is implemented based on the closest distance principle. The information obtained from the laser radar-detected targets is utilized to enhance the accuracy of the target information detected by the Mobileye system. The results of this processing are subsequently verified using videos, ensuring the acquisition of reliable and precise real vehicle data. In order to optimize the application of the data to the model, certain adjustments are made when the device fails to detect forward targets. Specifically, the headway distance is set to the farthest effective distance detected by the device, while the relative speed is adjusted to match the maximum value of that variable in the database. Additionally, when the vehicle speed reaches 0, the time headway (THW) is modified to correspond to the maximum value of that variable in the database.

The variables, excluding vehicle speed, have been classified into three categories based on their data sources. The first category comprises driving environmental information obtained from Mobileye or LiDAR. The second category includes vehicle operating state information gathered from the Inertial Navigation Unit (INU). The third category encompasses driving operation information collected via On-Board Diagnostics (OBDs). The purpose of this categorization is to examine the influence of vehicle surroundings, vehicle posture, and driving operations on risk prediction. Table 1 presents the specific categorization, variable descriptions, and symbolic information. Furthermore, in analyzing driving risk data, aggregated features were utilized over a specific time period. These features provide statistical analysis advantages for comprehensive data mining. The study calculated the upper quartile, lower quartile, maximum, minimum, mean, range, and standard deviation within the prediction window. Consequently, a total of 98 feature variables were generated (14 × 7).

## 3. Materials and Methods

This study utilized a significant volume of natural driving data to extract risk events, which were then employed for training and validating the risk prediction model. Initially, the risk indicator threshold was calibrated using the Monte Carlo simulation method. Subsequently, the risk events were identified using the threshold method and further validated and scrutinized through on-site video analysis. The feature variables incorporated in the model encompass diverse aspects of the driving process, encompassing driver control parameters, vehicle operating state parameters, and driving environment. To analyze the impact of various factors such as prediction window lengths, lead lengths, and data sources on the prediction performance, four machine learning algorithms were employed.

The issue of sample imbalance was addressed by implementing the random undersampling method. Optimal features were selected using the LGBM-Boruta method, and model parameters were optimized through Bayesian tuning. The most effective model was chosen for short-term driving risk prediction, and the causal analysis was conducted based on the prediction results.

### 3.1. Driving Risk Identification Method

In this study, braking deceleration and TTCi were used as indicators of driving risk events and combined with the risk quantification method proposed by Shangguan [15] to propose a risk indicator threshold calibration method based on Monte Carlo simulation. This method simulates the random deceleration of the front vehicle, and after the driver’s reaction time, the rear vehicle makes the maximum braking deceleration to determine whether a collision occurs. The random disturbance of the front vehicle follows a shifted gamma distribution *Ga* (17.32, 0.13, 0.66) [23], the driver’s reaction time follows a log-normal distribution *logN* (0.92, 0.28) [24], and the maximum braking deceleration of the rear vehicle follows a truncated normal distribution 4.23 ≤ N (8.45, 1.4) ≤ 12.68 [25]. Through 10,000 simulations, the risk threshold is calibrated based on the simulation results. Risk events are subsequently extracted using the threshold method and verified through video analysis. The algorithm for extracting driving risk events is outlined in Algorithm 1.

**Figure 2:**
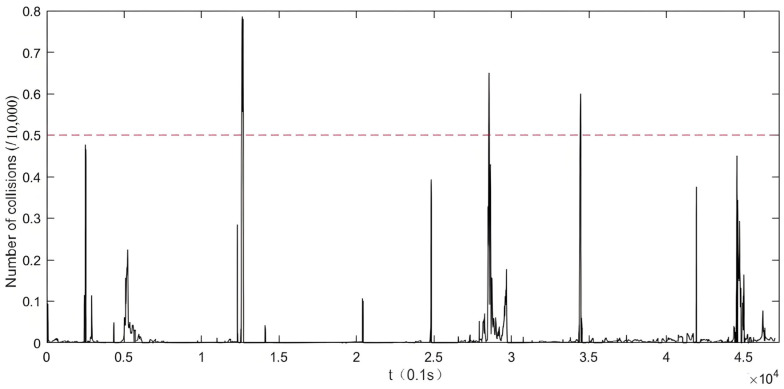
Carlo simulation results of partial driving fragments of driver 4.

Monte Carlo simulation is employed to estimate the probability of collision under car-following conditions, which serves as the basis for identifying preliminary driving risk states. As illustrated in Figure 2, Driver #4 is taken as an example. For each frame of driving data, a simulation is conducted to calculate the corresponding collision probability. When the probability exceeds 0.5, the moment is labeled as a preliminary risk state. For each risk indicator under this state, the distribution of its values is analyzed: for indicators where higher values indicate greater risk, the 85th percentile is extracted; for indicators where lower values indicate greater risk, the 15th percentile is extracted. These percentile values are used as reference thresholds to assess whether a given indicator significantly contributes to the identified risk state.

### 3.2. Variable Sliding Window and Negative Sample Downsampling Method

Risky driving data refers to the data collected within a specific time frame, specifically a few seconds before a risk event occurs. In this context, γ represents the lead length of the prediction window, while β denotes the length of the prediction window. The remaining time outside this window is categorized as normal driving data. To capture the evolving pattern of risks leading up to an event, a variable sliding window is utilized. This window adjusts its length based on the value, allowing for a comprehensive exploration of the data. Since there is a significant imbalance between risky and normal driving data, it is necessary to address this issue during dataset construction. To tackle the problem, random undersampling of negative samples is employed. Specifically, normal driving data are divided into fixed-length driving segments in sequential order. Prior to the maximum risk window width of each segment, a random number is generated. Subsequently, the data extracted from the window, which has a width of β units after the random number, are considered as negative samples. This approach effectively reduces the number of negative samples, while still retaining most of the information from the normal driving data. Consequently, it successfully resolves the challenge of data imbalance in risk prediction. Overall, this approach allows for a thorough analysis of risky driving data and normal driving data, while addressing the data imbalance issue, thus enhancing the accuracy of risk prediction.

**Figure 3:**
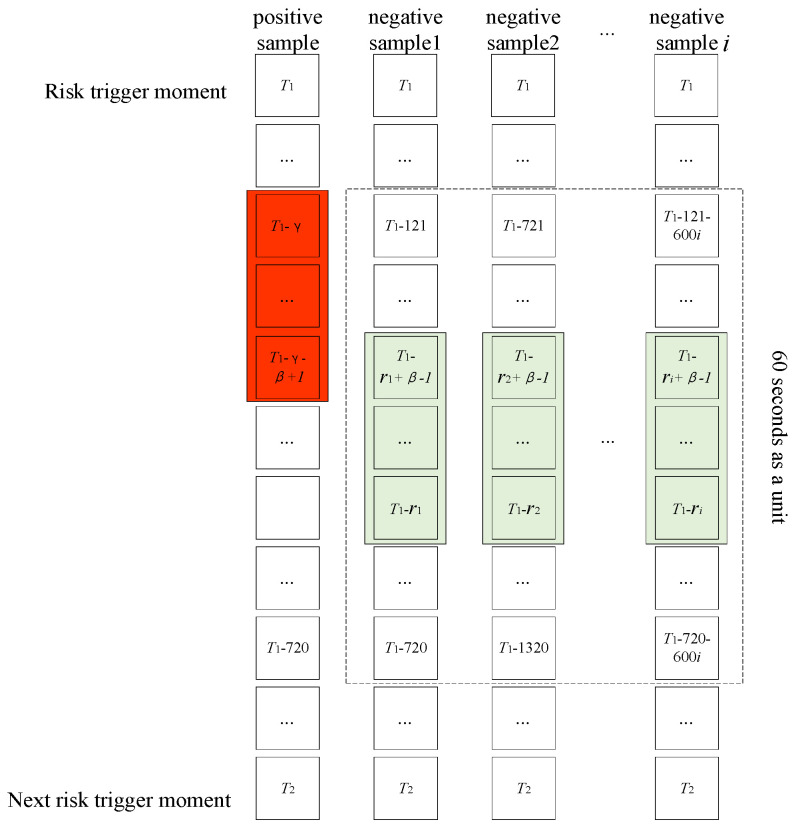
Positive and negative sample extraction methods.

The method described in Figure 3 was implemented using a fixed length of 60 s. With this configuration, the ratio of positive to negative samples extracted was found to be 942:3533. This ratio adheres to the general model sample balance condition. A total of 676 datasets were generated for model input by extracting data with a step size of 0.2 s from prediction window lead length γ ∈ (1,6) s and prediction window length β ∈ (1,6) s.
**Algorithm 1** Risk status identification algorithm**Input**: vehicle speed $v_{t}$, front vehicle speed $v_{f}$, distance between two cars $R_{d}$, reciprocal of TTC TTCi, braking deceleration BD N←0,k←1 **for** i←1 **to** F **do** **for** i←1 **to** 10,000 **do** $\begin{array}{l} D_{f}\leftarrow gamrnd(17.3,0.13,0.66)-0.66\\ R_{t}\leftarrow \log nrnd(0.92,0.28)\\ D_{M}\leftarrow 4.23\leq normrnd(8.45,1.4)\leq 12.68 \end{array}$ **if** $v_{f}^{2}/\left(2*D_{f}\right)+R_{d}\leq v_{t}^{2}/2*D_{M}$ **then** N→N+1 **end** **end** **if** N≥5000 **then** $TTCi\left(k\right)\leftarrow TTCi,BD\left(k\right)\leftarrow BD,k\leftarrow k+1$ **end** **end** $Th_{TTCi}\leftarrow median\left(TTCi\right),Th_{BD}\leftarrow median\left(BD\right)$ **for** i←1 **to** F **do** **if** $TTCi\left(i\right)\geq Th_{TTCi}\left|BD\right.\left(i\right)\leq Th_{BD}$ **then** $Sign_{risk}\left(i\right)\leftarrow 1$ **end** **end** **Output**: Risk label (0,1)

### 3.3. LightGBM-Boruta Feature Selection Method

A LightGBM-Boruta feature selection method is proposed. This method uses the feature importance output from the LightGBM model to screen out the feature set that is correlated with the dependent variable. To accomplish this, a shadow feature S is created to correspond with each real feature R. Subsequently, a new feature matrix [R, S] is formed, serving as the input for the LightGBM model. The feature importance of each feature is then calculated using the following procedure:

The entropy *Entropie*(*D*) of the sample set in a tree node is as follows:

(1) $$Entropie(D)=-\sum_{k=1}^{K}\frac{\left|C_{k}\right|}{D}log\frac{\left|C_{k}\right|}{D}$$

If the number of times the sample set is divided according to feature A is n, the feature importance *Z_A_* is as follows:

(2) $$Z_{A}=(\sum_{i=1}^{n}Entropie(D)-\;Entropie(D|A))/n$$

**Figure 4:**
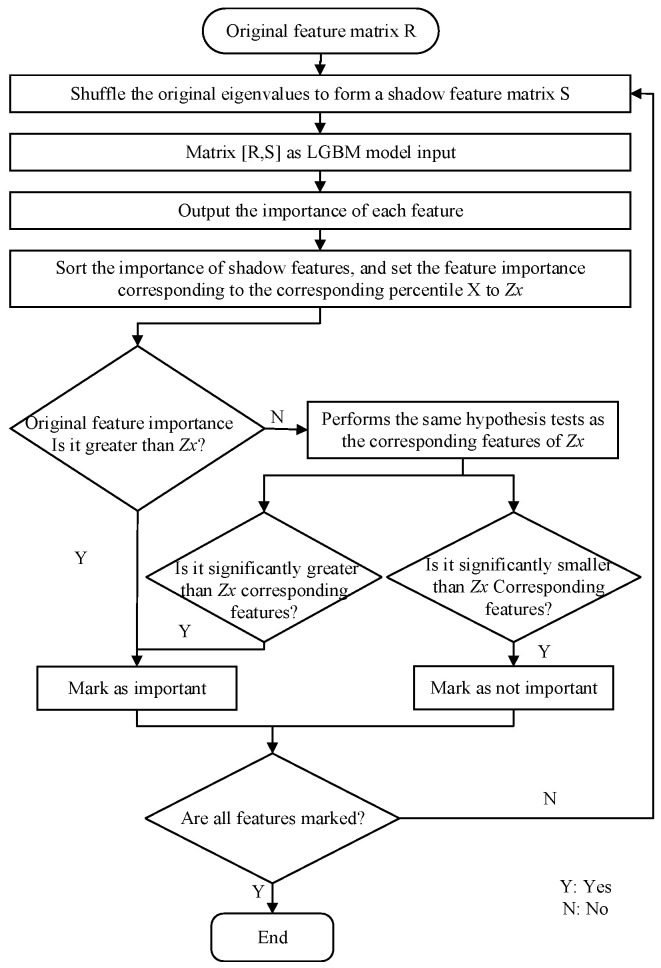
Feature selection flowchart.

The shadow features are sorted based on their importance, and a percentile value is selected as the threshold for comparing the importance of real features. If the importance value of a real feature exceeds this threshold, it is considered significant and selected for further analysis. A two-sided test is then conducted to eliminate shadow features whose importance is significantly lower than the corresponding percentile. This process continues iteratively until all features have been marked with their respective importance. The specific algorithm flow chart is shown in Figure 4.

### 3.4. Driving Risk Prediction Model

The short-term prediction model for driving risk aims to detect abnormal driving data shortly before a risk event occurs. It utilizes a classification model to identify segments of driving data that deviate from normal patterns. By employing data processing techniques mentioned earlier, such as combining natural driving data and employing various machine learning methods, namely logistic regression (LR), multilayer perceptron (MLP), extreme gradient boosting (XGBoost), and light gradient-boosting machine (LGBM), pre-classification is performed. These four classification algorithms, LR, MLP, XGBoost, and LGBM, were chosen based on their superior performance. The evaluation of the model’s performance includes accuracy rate, recall rate, precision rate, and F1 value as indicators.

(1) LR:

The LR algorithm is widely recognized as one of the most commonly used and traditional algorithms in machine learning. It offers several advantages, including a simple model structure, easy interpretability, and fast training speed. By incorporating a sigmoid function into the linear regression model, the algorithm maps the predicted results to values between 0 and 1, facilitating categorical determination.

(2) MLP:

MLP is a highly popular artificial neural network algorithm utilized in various domains. It comprises three layers: the input layer, hidden layer, and output layer [26]. The input layer receives the necessary feature vectors, with each neuron fully connected to neurons in the preceding hidden layer. The hidden layer is so named due to its lack of direct interaction with the external environment. Neurons in both the hidden and output layers are fully interconnected, and the model’s prediction results are generated accordingly.

(3) XGBoost:

XGBoost, initially proposed by Dr. Chen, has emerged as a prominent integrated machine learning algorithm renowned for its exceptional characteristics and interpretability. Based on the CART (Classification and Regression Tree) model, XGBoost employs multiple CART trees in collaboration [27]. The CART tree selects features and optimal splitting points by minimizing the Gini index, and it undergoes pruning to prevent overfitting. By adopting the concept of Gradient Tree Boosting, XGBoost enables ensemble learning of multiple CART trees. This entails calculating the negative gradient of the objective function to guide the optimization of submodels.

(4) LGBM:

LGBM shares fundamental principles with XGBoost and serves as an implementation of gradient-boosting decision trees. However, it distinguishes itself with its emphasis on being “lightweight,” achieving a faster runtime and reduced memory consumption. LGBM enhances the splitting point and leaf node growth strategies employed in the XGBoost algorithm. It organizes feature values by size and utilizes histograms to store continuous features in discrete bins, facilitating efficient search for optimal features and split points. LGBM offers two growth methods for leaf nodes: layer-by-layer and leaf-by-leaf [28]. Unlike XGBoost’s layer-by-layer approach, LGBM’s leaf-by-leaf growth method identifies nodes with higher information gain for expansion, thereby enhancing prediction accuracy while preventing excessive model complexity. To control tree depth, LGBM employs a leaf-by-leaf growth algorithm with depth constraints.

### 3.5. SHAP

The SHAP method was improved by Shapley in 1953 in combination with cooperative game theory to solve the problem of revenue allocation after multiple players cooperate to complete tasks in a game. By leveraging cooperative game theory principles, SHAP offers a novel approach to measuring the impact of features on a model. This is accomplished by quantifying the changes in output resulting from the absence of specific features across all possible feature combinations. By leveraging cooperative game theory principles, SHAP offers a novel approach to measuring the impact of features on a model. This is accomplished by quantifying the changes in output resulting from the absence of specific features across all possible feature combinations. The prediction result of the model is obtained by adding the SHAP values, which can be expressed as follows:

(3) $$g(z)={Ø}_{0}+\sum_{i=1}^{M}{Ø}_{i}*z$$

where ${Ø}_{0}$ is a bias term that typically predicts the mean for all samples. ${Ø}_{i}$ represents the SHAP value of characteristic variable *i*, $z\subseteq 0,1^{M}$, and *M* is the characteristic quantity. The characteristic variable *z* for the selected analysis is 1; otherwise, it is 0.

(4) $${Ø}_{i}=\sum_{S\subseteq N\backslash \left\{i\right\}}\frac{\left|S\right|!(M-\left|S\right|-1)!}{M!}[f_{x}(S\cup i)-f_{x}(S)]$$

*S* represents a subset of all feature variables set *N* except for *i* feature variables, $\left|S\right|!$ represents the number of permutations and combinations before feature in the sequence, and $f_{x}(S\cup i)$ and $f_{x}(S)$ represent the predicted output and expected output of a given set of *S* except for the *i* characteristic variable, respectively.

## 4. Results and Discussion

### 4.1. Risk Evolution Law

Four machine learning models were employed to predict short-term driving risks using a variable sliding window approach. Although model performance has been enhanced to some extent through feature selection and parameter tuning, there is currently no definitive method to identify the absolute best parameters. Tuning each model individually may impede the exploration of risk evolution patterns. Thus, the primary objective at this stage is to determine the optimal risk trigger lead time and risk window length. Consequently, all feature variables were utilized as inputs for the models without any parameter tuning. To mitigate the influence of randomness in individual predictions, the training set and validation set were randomly divided into ten subsets, and the validation results were averaged. The F1 score was employed to ascertain the optimal lead length and length of the prediction window.

**Figure 5:**
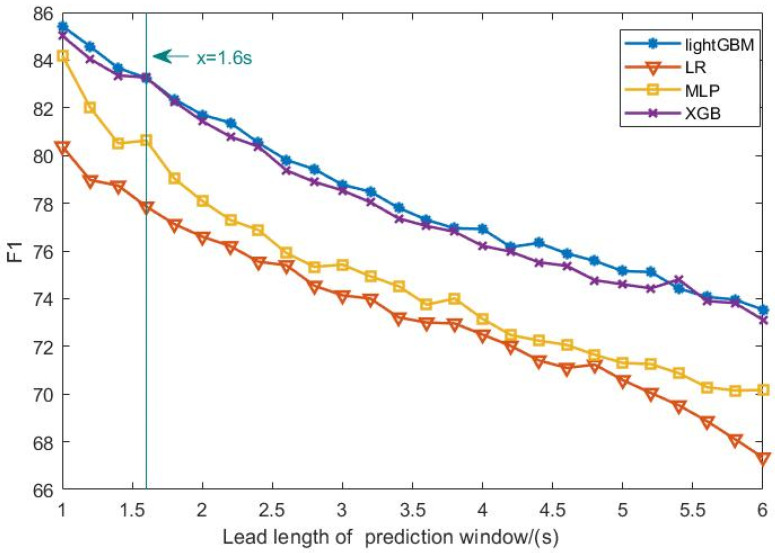
Four models predict F1 values under different prediction window lead lengths.

Figure 5 illustrates the relationship between the prediction window length and the prediction performance (measured by F1 score) of different machine learning models. As expected, the predictive performance generally declines as the window length increases, although the rate of decline differs across models. Notably, when the prediction window length reaches 1.6 s, all models experience a temporary improvement in predictive ability, followed by a sharp decline. Among them, the XGBoost and LGBM models demonstrate the best overall performance, with the optimal prediction window length determined to be 1.2 s. Therefore, the optimal prediction lead time and window length are established as 1.6 s and 1.2 s, respectively.

In Figure 5, the horizontal axis represents the prediction window length (in seconds), while the vertical axis indicates the F1 score on the validation set. A higher F1 score signifies better predictive performance. The curves in the figure illustrate how each model’s performance varies with different window lengths. It is evident that different models respond differently to changes in window length, with tree-based models exhibiting notably superior performance at specific lengths.

Additionally, as shown in Table 2, each model has distinct requirements for data aggregation, and the choice of window length is closely related to the underlying classification mechanisms and features used. For example, logistic regression (LR), as a linear model, performs better at longer window lengths. In contrast, the multilayer perceptron (MLP), which has strong nonlinear fitting capabilities, does not display a clear optimal window pattern. For XGBoost and LGBM, the optimal window length is relatively short when the prediction lead time is less than 5 s, but becomes longer when it exceeds 5 s. This suggests that the window length should be adapted to the specific prediction time horizon to fully leverage the model’s performance.

### 4.2. Feature Selection Results

The selection of features in this study was carried out using the LGBM-Boruta method, with a prediction window lead length of 1.6 s and a prediction window length of 1.2 s. To determine the most relevant features, shadow feature importance scores were employed as thresholds at various percentile values. The evaluation involved calculating the number of selected feature variables and the F1 value predicted by LGBM on the test set across different threshold values.

**Figure 6:**
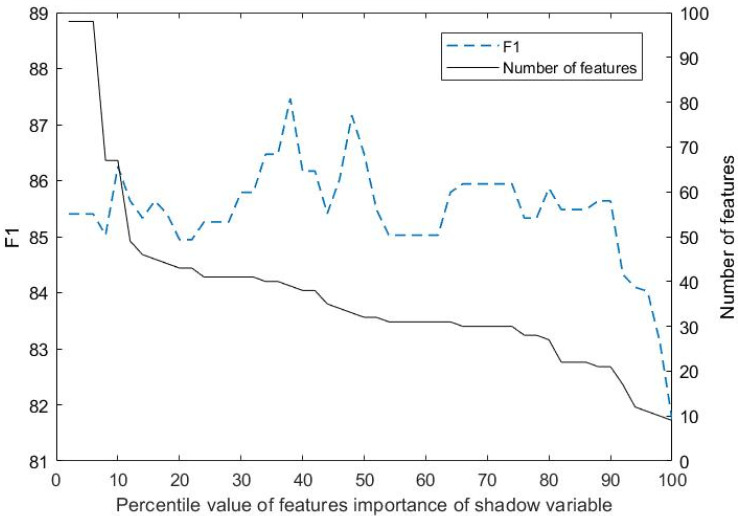
The number of feature selections and predicted F1 values under different thresholds.

Figure 6 illustrates the relationship between the threshold and the number of selected feature variables. In the figure, the x-axis represents the percentile threshold of shadow feature importance. The dashed line indicates the F1 score, while the solid line represents the number of selected feature variables. It is evident that as the threshold increases, the number of selected feature variables gradually decreases. When the selection criteria are the most stringent, only nine feature variables (VEL_Mean, VEL_SD, THW_Min, THW_Range, RTC_Max, LAC_Min, LAC_SD, GPD_Mean, STA_SD) are chosen, indicating their strong correlation with the risk status. Additionally, it was observed that when the number of characteristic variables falls below 20, the prediction performance of the model declines rapidly. The combination of characteristic variables corresponding to the maximum F1 value was selected. Consequently, a total of 39 characteristic variables were chosen as inputs for the model. The detailed results are presented in Table 3.

### 4.3. Model Prediction Results and Comparison

Given the wide application and good performance of logistic regression (LR), multilayer perceptron (MLP), and extreme gradient boosting (XGBoost) in risk prediction, this study also uses these algorithms to conduct short-term risk prediction based on the dataset constructed in this research [26,27,28]. The results of these algorithms are then compared with those of the improved light gradient-boosting machine (LGBM) in this study, and the comparison is presented in Table 4.

It was observed that feature selection significantly improved the performance of all models except for XGBoost, particularly in terms of recall. The increase in recall indicates a higher probability of correctly predicting all true risk events. However, the precision of the models decreased, indicating a lower probability of correctly predicting all predicted risk events. Among the models, LR exhibited the highest precision but the lowest recall, with values of 89.17% and 71.10% respectively. This indicates that the LR model is sensitive to specific risk situations, but it is highly reliable for those situations. On the other hand, the LGBM model outperformed all other models, displaying the highest recall and good precision. The LGBM model achieved a remarkable accuracy of 90.20% in predicting all risk events within 1.6 s, with a predicted credibility of 85.71%.

Based on the information presented in Table 5, the LGBM model with the highest predictive performance was utilized to make short-term predictions for three distinct data sources. These data sources are interconnected in a cause-and-effect relationship. Changes in the surrounding environment influence driver manipulation, which subsequently affects the vehicle’s attitude. However, it is worth noting that there is a slight time delay in the response of driver manipulation. Additionally, driver manipulation data lag behind changes in the surrounding environment due to the driver’s reaction time. Consequently, the predictive performance of the three data sources follows an increasing order, with the highest performance achieved by the human–vehicle–road environment information. This is attributed to the comprehensive nature of this data source, encompassing all relevant factors. Remarkably, the predictive performance of all data sources, including the human–vehicle–road environment information, significantly outperforms the first three sources alone, as indicated by an impressive F1 score of 87.90%.

### 4.4. SHAP Analysis

**Figure 7:**
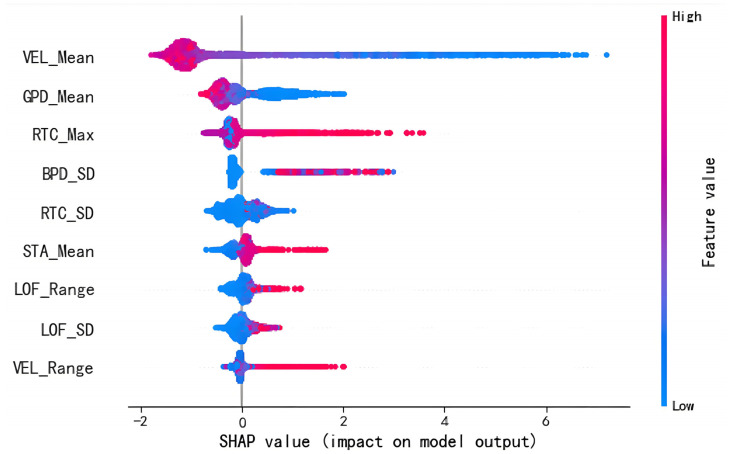
SHAP analysis summary diagram.

After conducting feature selection using all available data, SHAP analysis was performed on the trained LGBM model to examine the impact of lead length and prediction window length within the optimal prediction window. The outcomes of this analysis are illustrated in Figure 7.

As shown in Figure 7, the plot depicts several key feature variables that significantly influence the short-term driving risk prediction model. The x-axis represents the SHAP value, where a larger positive value indicates a stronger contribution to the prediction of a positive (risky) label, and a more negative value indicates a stronger contribution to a negative (non-risky) label. The color of each point reflects the actual value of the corresponding feature: dark blue indicates a lower value, and red represents a higher value. Each point in the plot corresponds to a specific feature of a sample in the dataset.

The analysis reveals that VEL_Mean, GPD_Mean, and RTC_Max are significant factors in risk prediction, indicating the importance of speed, throttle position, and TTCi in ADAS risk perception. Notably, approximately 1.6 s prior to a risk event, the vehicle speed typically decreases to a lower level. Surprisingly, an increase in VEL_Mean corresponds to a decrease in risk, which could be attributed to a reduced number of risk samples occurring at higher speeds on highways with less complex road conditions and lower traffic volumes. Furthermore, a smaller GPD_Mean and a larger RTC_Max signify a higher risk level, suggesting that most drivers are aware of impending risks and take necessary braking measures. Additionally, an increase in STA_Mean and LOF_Range is associated with an elevated risk, indicating a greater likelihood of risk during lane changes or turns compared to other driving scenarios. Therefore, understanding the significance of these feature variables can aid vehicle manufacturers and transportation departments in comprehending driver behavior and devising effective safety measures to mitigate the risk of traffic accidents.

## 5. Conclusions

This study presents a novel approach for short-term driving risk warning based on onboard sensing data. The key contribution of this paper is the introduction of a new method to calibrate the risk index threshold and determine the risk event identification method. The study incorporates various techniques such as a variable sliding window, negative sample undersampling, LGBM-Boruta feature selection, and classic machine learning to develop a short-term prediction system for driving risks. The main findings of the study are summarized as follows:

(1) The proposed risk event identification method establishes the risk thresholds for TTCi and braking deceleration as 0.86 s^−1^ and −2.6 m/s^2^, respectively.
(2) A combination of four machine learning methods and a variable sliding window approach is employed to explore the pattern of risk evolution. The study reveals that as the prediction window lead length increases, the prediction performance generally decreases, but the decline rate varies. The optimal prediction window lead length and prediction window length are determined as 1.6 s and 1.2 s, respectively, based on comprehensive considerations.
(3) The short-term driving risk prediction model utilizes the statistical Boruta method for feature selection. This technique effectively filters out irrelevant feature variables, simplifies the model’s input variables, and improves risk prediction performance.
(4) Among the models evaluated in this study, the LR model exhibits the highest accuracy at 89.17%, while the LGBM model achieves the best recall and F1 values at 90.20% and 87.90%, respectively. This method demonstrates its potential to meet the risk prediction requirements of future ADAS systems. The prediction performance varies depending on the data sources, with the following ranking in terms of effectiveness: surrounding information > driver manipulating information > vehicle attitude information. Notably, combining data from all three sources significantly enhances the prediction performance compared to individual data source predictions.
(5) SHAP analysis identifies VEL_Mean, GPD_Mean, and RTC_Max as the most influential features impacting short-term driving risk prediction. Some of these features also serve as crucial indicators for distinguishing different driving conditions. Future work can focus on dividing driving conditions initially and then conducting training and prediction specific to each condition, leading to improved prediction results.

In this study, we developed a high-grade roads dataset to verify the effectiveness of our risk prediction methodology using in-vehicle perception data under high-grade road conditions. When constructing the dataset, we incorporated various types of high-grade roads, including urban arterial roads, urban expressways, and freeways, and took different speed limits into account. The resulting dataset demonstrates good generalizability for the short-term risk prediction method based on in-vehicle perception data on high-grade roads. To further strengthen the credibility of the model’s performance, conducting tests in actual vehicles equipped with ADASs is advisable. Building on this, future research proposes to conduct experiments with a simulated or real warning system. These experiments aim to assess drivers’ reactions to the predicted risk, which will provide more practical insights into the effectiveness of our prediction method.

However, the applicability of this method in low-grade roads, areas with special road features (such as sharp curves, long and steep slopes), and intersections still needs to be verified. In future research, we intend to use datasets from other scenarios to test the effectiveness of this method. Based on these tests, our goal is to further enhance the method and develop an algorithm with greater generalization ability. Moreover, considering external factors such as pedestrian and cyclist behavior or dynamic changes in traffic can contribute to improving the accuracy of short-term risk prediction. In light of this, we plan to further refine the dataset, which will not only improve the prediction accuracy but also help us better understand how the method performs in different real-world situations. This continuous improvement process will enable us to develop a more robust and reliable risk prediction system.

## Figures and Tables

**Table 1:** Explanation of the features during the lane change.

Devices	Variables	Variables Description	Symbols
	Velocity	Vehicle speed, km/h	VEL
Mobileye + Lidar (surroundings)	Lane curvature	Lane line curvature, $m^{-1}$	LCU
	Lane offset	The distance from the center of the vehicle to the centerline of the lane, m	LOF
	Relative distance	Distance from the vehicle ahead, m	RDS
	Relative velocity	Relative velocity from the vehicle ahead, km/h	RVS
	THW	Time headway, s	THW
	Reciprocal of TTC	Reciprocal of time to collision, s^−1^	RTC
IMU (vehicle attitude)	Longitudinal acceleration	Vehicle longitudinal acceleration, m/s^2^	LAC
	Later acceleration	Vehicle lateral acceleration, $m/s^{2}$	LTA
	Yaw rate	Vehicle yaw rate, rad$/s^{2}$	YRT
OBD (driver maneuvering)	Gas pedal	Throttle opening, %	GPD
	Brake pedal	Brake pressure, Mpa	BPD
	Steering angle	Steering wheel angle, rad	STA
	Steering distance	Steering wheel angle speed, rad/s	STD

**Table 2:** Optimal prediction window length under different prediction window lead lengths.

Prediction Window Lead Length (s)	LR (s)	MLP (s)	XGB (s)	LGBM (s)
1.0	4.4	1.0	2.4	1.0
1.2	4.0	1.0	1.2	1.2
1.4	5.8	1.0	1.2	1.2
1.6	5.6	1.0	1.2	1.2
1.8	5.8	1.0	2.4	3.2
2.0	4.8	1.0	2.2	1.8
2.2	5.0	1.0	1.4	1.0
2.4	4.6	4.6	2.2	2.2
2.6	4.4	4.0	2.8	2.2
2.8	4.2	4.2	1.6	1.8
3.0	4.0	4.2	1.8	1.0
3.2	4.0	1.2	1.4	1.4
3.4	4.2	2.0	1.0	1.6
3.6	5.4	1.6	1.4	1.8
3.8	4.8	1.0	1.0	1.6
4.0	5.2	1.0	1.6	1.2
4.2	4.8	5.0	1.0	1.4
4.4	4.2	5.0	1.2	1.2
4.6	4.6	5.0	1.0	1.0
4.8	4.2	4.8	5.4	1.0
5.0	4.2	5.0	4.6	1.0
5.2	4.2	5.0	5.4	4.8
5.4	4.2	4.6	4.6	5.2
5.6	3.8	4.6	5.2	4.6
5.8	4.4	2.2	3.6	3.6
6.0	4.6	4.0	3.6	5.4

**Table 3:** Feature filtering results for maximum F1 value and maximum threshold.

Symbols	Features						
	UQ	LQ	Mean	Max	Min	Range	SD
VEL	-	-	●	-	-	○	●
LCU	-	-	○	-	-	-	-
LOF	○	-	-	-	○	○	○
RDS	-	-	-	-	-	-	-
RVS	-	○	-	-	-	-	-
THW	-	○	○	○	●	●	○
RTC	-	○	-	●	-	○	○
LAC	○	-	-	○	●	○	●
LTA	-	-	○	○	-	-	○
YRT	-	-	○	-	-	○	○
GPD	-	-	●	-	-	○	○
BPD	-	-	-	-	-	○	○
STA	-	-	○	-	-	○	●
STD	-	-	○	-	-	-	-

Note: ○ indicates selected feature at the maximum F1 value, ● indicates selected feature at the maximum threshold value.

**Table 4:** Prediction effect of different optimization models before and after feature screening.

Models		Accuracy	Recall	Precision	F1 Score
LR	I	91.65	66.45	**90.91**	76.78
	II	92.2	71.1	89.17	79.11
MLP	I	92.69	76.08	87.07	81.21
	II	92.2	89.17	80.07	83.25
XGB	I	94.07	82.39	88.27	85.22
	II	94.14	82.39	88.57	85.37
LGBM	I	94.28	82.06	89.49	85.62
	II	**95.1**	**90.2**	85.71	**87.9**

Note: I indicates the result before feature selection, II the result after feature selection.

**Table 5:** Prediction effect of LGBM model under different data sources.

Models		Accuracy	Recall	Precision	F1 Score	Number of Features
Driver manipulating	I	91.92	75.22	84.61	79.59	35
	II	91.92	75.22	84.61	79.59	15
Vehicle attitude	I	91.74	73.41	85.16	78.82	28
	II	91.87	73.67	85.54	79.13	13
Surroundings	I	92.44	77.98	84.81	81.2	49
	II	92.47	77.77	85.08	81.22	18
All	I	94.28	82.06	89.49	85.62	98
	II	95.1	90.2	85.71	87.9	39

Note: I indicates the result before feature selection, II indicates the result after feature selection.

## Data Availability

Data are contained within the article.
